# Photic Phase Shifts of the Mouse Circadian Clock Are Defined by Scene Irradiance Across Differences in the Spatial Distribution of Light

**DOI:** 10.3390/clockssleep8040062

**Published:** 2026-09-29

**Authors:** Qian Huang, Franck P. Martial, Riccardo Storchi, Robert J. Lucas

**Affiliations:** 1 Centre for Biological Timing, School of Biological Sciences, Faculty of Biology, Medicine and Health, University of Manchester, Manchester M13 9PT, UK; 2 Division of Neuroscience, School of Biological Sciences, Faculty of Biology, Medicine and Health, University of Manchester, Manchester M13 9PT, UK

**Keywords:** circadian rhythms, light, irradiance, spatial distribution, phase shifts, mice, patterned illumination, circadian photoreception

## Abstract

Light resets the mammalian circadian clock, but whether this response is determined solely by integrated light intensity (irradiance) or is also influenced by the spatial distribution of light remains unclear. We tested whether high-contrast or low-spatial-frequency patterns modulate circadian responses in mice independently of scene irradiance. Circadian phase shifts were measured following exposure to spatially patterned illumination, including high-contrast checkerboards and spot stimuli positioned at the horizon or zenith. Across all experiments, redistributing light within the visual field did not measurably alter phase-shift amplitude when irradiance was matched between paired conditions. Circadian responses were well predicted by total scene irradiance whereas a metric based on the region of highest radiance provided a poorer account of the data. These findings are consistent with effective spatial integration of light by the mouse circadian system. Within the range of spatial and irradiance conditions tested, our results support irradiance as a simple and effective metric for predicting circadian responses across light environments with divergent spatial distributions.

## 1. Introduction

The mammalian master circadian clock in the hypothalamic suprachiasmatic nuclei (SCN) is set to local time (photoentrained) by input from a subset of intrinsically photosensitive retinal ganglion cells (ipRGCs) [1,2]. In both experimental and applied branches of circadian biology the activity of this photoentrainment pathway is typically assumed to reflect ambient light as quantified by measuring irradiance (or illuminance, its photometric equivalent). The irradiance metric quantifies summed light falling on a detector surface from all directions according to a cosine-corrected angular sensitivity. In effect, then, describing an environment in terms of irradiance incorporates the assumption that the spatial distribution of light within the scene has no importance (except insofar as it makes the outcome dependent on the direction in which the detector is pointed). This practice reflects the fact that the change in environmental irradiance as a function of solar angle has hard-to-beat signal:noise for telling time of day. Moreover, innumerable studies across many species have shown that changing irradiance without altering its spatial distribution modifies circadian photoresponse. However, the ipRGCs innervating the SCN exist within an optical apparatus (the eye) that ensures that they each collect light from a restricted portion of the field of view [3], meaning that each individual detector unit does not match the properties of an irradiance detector. Accordingly, ipRGCs have an ability to encode spatial patterns that is inherited by the SCN [4,5]. This raises the question of whether spatial distribution is an additional determinant of the circadian clock’s response to light.

Perhaps the strongest experimental evidence in favour of ignoring spatial patterns when quantifying light comes from studies recording circadian responses to full field light pulses of different duration and interval. These establish that the mammalian circadian system integrates visual input over up to many minutes [6,7]. Within such timeframes, shifts in direction of view would minimise the significance of spatial patterns, such that the circadian response could reflect features of the whole light environment. However, temporal integration in photoentrainment is imperfect, and can fail for stimuli directed towards cone photoreceptors and at fine timescales [8,9,10,11]. Additionally, it is formally possible that the uncoordinated modulation in neuronal firing across the ipRGC population caused by shifting direction of view across spatial patterns may have a different impact on circadian rhythms to the stylised and coordinated modulation induced by changing full field illumination. Furthermore, there is both experimental and anatomical data indicating that some parts of the retina may make a disproportionate contribution to the light signal reaching the circadian system [12,13,14,15], implying that under natural view some portions of the scene (e.g., ground or sky) could be especially significant in defining circadian responses. Finally, the maintained firing rate of at least some ipRGCs (which encodes irradiance) is additionally impacted by visual contrast [16].

These considerations highlight the need for direct experimental assessment of the significance of spatial patterns for circadian light responses. A previous study showed that introducing drifting vertical gratings modestly enhanced time averaged firing in the SCN of anaesthetised mice compared to irradiance-matched featureless stimuli but did not have a significant impact on circadian phase shift magnitude in awake animals [4]. That work argues that under many circumstances, the spatial distribution of light is not relevant for predicting the clock response. However, the patterned stimulus employed in that work did not capture all features of natural environments. Firstly, because those stimuli were presented using a liquid crystal display, they could not encompass the very large differences in radiance that can be encountered across natural scenes [17]. Secondly, the use of drifting vertical gratings fails to capture one of the most characteristic features of natural scenes, the brightness gradient as a function of elevation [18]. The latter is a particular consideration, both because ipRGCs innervating the mouse SCN appear more densely in the dorsal retina and because vertical light distribution may reflect time of day thanks to its potential dependence on solar elevation. Here we therefore set out to ask whether either very high contrast patterns or differences in vertical light distribution impact circadian resetting in mice.

## 2. Results

We designed a chamber in which it would be possible to present spatial patterns of defined characteristic to mice. A transparent container (15 cm diameter) was placed inside a larger diffusive (opal Perspex) box (75 × 75 × 110 cm) lit from the outside by an array of white and violet light-emitting diodes (LEDs) arranged to present near uniform illumination to the inside of the box (Figure 1A). Spatial patterns could then be introduced either by switching off some of the lights or placing a mask on the outside of the outer chamber. We started by constructing an intensity response curve for phase shifts in the circadian locomotor activity rhythm in response to spatially uniform illumination presented in this apparatus. Mice housed in a 12:12 light:dark (LD) cycle were transferred to the apparatus for 15 min, 4 h after activity onset (designated zeitgeber time 16 (ZT16)) and released into constant darkness. As expected, this light pulse produced a phase delay in the circadian activity rhythm (Figure 1B) whose magnitude was positively correlated with stimulus irradiance (Figure 1C). The resultant irradiance response curve could be approximated with a sigmoidal function (R^2^ = 0.63) with half-maximal response at a melanopic equivalent daylight (D65) illuminance (melanopic EDI) of 3.29 lx, somewhat higher than previously reported for equivalent light pulses presented to animals held for several days in darkness [4,19,20].

Having identified an irradiance range capable of inducing sub-saturating phase shifts, we continued to generate pairs of stimuli matched for irradiance within this range but presented either as a featureless illumination or with a checkerboard spatial pattern. We applied masks to the outside surface of the arena to generate checkerboard patterns covering the scene above the horizon (Figure 2A), with a full cycle in the horizontal dimension occupying 22.5° of the visual field from the centre of the arena (spatial frequency of 0.044 cycles per degree (cpd)), well within the spatial resolution of mouse vision [21,22]. Radiance in dark portions of the pattern was >1000× (×10^−3.6^) dimmer than in the bright. We generated two checker stimuli differing in the relative width of dark and bright regions (checker 1 = dark portions 3× wider than bright, checker 2 = dark portions 7× wider). As bright phase radiance was retained across the 2 checkers (12.32 log_10_(melanopsin-effective photons cm^−2^ s^−1^ sr^−1^)), including both allowed us to probe responses at two irradiances (4.16 and 2.10 lx melanopic EDI for checkers 1 and 2 respectively) falling on the steepest portion of the irradiance response curve. We then compared circadian phase shifts induced by 15 min exposure to either of these checkers vs. diffuse illumination at that irradiance. In neither case was there a significant difference in the magnitude of phase shift between checker and featureless illumination (Figure 2B; paired *t*-test: Checker 1, t(13) = 1.43, *p* = 0.18; Checker 2, t(9) = 0.85, paired *t*-test, *p* = 0.42).

We next turned our attention to lower spatial frequency patterns. In principle variations in light distribution across elevation may be more impactful both because ipRGCs are not uniformly distributed across the retina [23,24,25] and because solar elevation could be used to tell time of day. To test this possibility, we lit the arena with a diffuse spot placed either just above the horizon, such that the entire spot remained visible, or directly above the arena (Figure 2C). The light spot was created by a combination of violet and white light to approximate the mouse experience of the spectral quality of daylight. We found that equivalent magnitude circadian phase shifts were induced by 15 min exposure to the arena lit with a spot in either location (Figure 2D; Paired t test, t(12) = 0.26, *p* = 0.80).

The similarity in phase shift magnitude for environments of equivalent irradiance but differing spatial distribution of light argues that spatial patterns of the type presented here do not materially impact the circadian response to light. For comparison we finally tested an alternative hypothesis regarding the light parameter defining phase shift amplitude. In situations of diffuse illumination irradiance perfectly correlates with the radiance of the brightest region of the scene making it impossible to distinguish which of these parameters defines response amplitude. Our spatial patterns dissociate these two metrics. We therefore compared responses to our patterned stimuli with the intensity response relationship for diffuse light expressed in terms of peak radiance (Figure 2E). Mean phase shift amplitude for checker 2 and both spot stimuli fell outside the 95% confidence interval for the radiance response curve for diffuse illumination (Table 1) confirming that peak radiance is not an adequate predictor of circadian response.

## 3. Discussion

We set out to ask whether either high contrast or low frequency spatial patterns impact the circadian response to a light environment. We find that changing the spatial distribution of light did not impact circadian response amplitude. This was true for both high contrast checker patterns and for spot illumination at horizon vs. zenith.

How confident can we be that the spatial distribution of light never has significance for mouse circadian responses? Leaving aside the possibility of small differences in response amplitude below the resolution of our methods, the answer to this question depends to a large extent on the degree to which our stimuli cover patterns a mouse may reasonably expect to encounter. The within scene radiance range of our patterned stimuli should exceed that found in most natural scenes [17]. Our spot stimuli cover the extremes of the location of brightest region in the vertical dimension associated with changes in solar elevation (although no laboratory stimulus can recreate the point source radiance of direct sunlight). It seems likely, therefore that our conclusions are applicable for most naturally occurring patterns in brightness. On the other hand, we have not explored the significance of patterns in colour. The spectral composition of our stimuli was designed to recreate the mouse experience of daylight but not of spatial variations in spectrum that may show time of day dependence [26]. Moreover, none of our scenarios cover light intensities that can be expected in daytime. It may be that spatial summation rules are different under very bright ambient light or in the presence of an extremely bright spot source like the sun. However, as circadian responses in mice generally saturate at twilight irradiances that feels more a theoretical than practical consideration.

How generalisable are these findings beyond adult male C57BL/6J mice? Sex and hormonal status can influence photic phase shifting and re-entrainment [27,28,29,30,31,32], while circadian light responses and melanopsin-positive cell topography vary among mouse strains [33,34,35]. Whether these factors affect spatial integration in photoentrainment is unknown. Temporal niche may also matter: melanopsin-immunoreactive cells are distributed relatively uniformly in nocturnal *Micaelamys namaquensis* but are concentrated dorsonasally in diurnal/crepuscular *Rhabdomys pumilio* [36]. This contrast could motivate hypotheses of broader pooling in the former and stronger regional weighting in the latter. However, in diurnal *Arvicanthis niloticus*, M1–M3 ipRGCs are relatively evenly distributed [37], while nocturnal laboratory mice also show regional differences among ipRGC populations [12,23,24,25]. Together, these findings indicate that temporal niche alone does not explain retinal distribution of ipRGCs. Whether such variation affects spatial summation, including whether localised stimulation can saturate circadian phase resetting, remains unknown and requires matched functional comparisons across sexes, genetic backgrounds and a variety of species.

How is spatial averaging accomplished? In non-mammalian species, spatial averaging may be a consequence of employing photoreceptors for photoentrainment that are themselves not sensitive to the direction of light. In invertebrates and non-mammalian vertebrates, circadian photoreceptors are found outside the eyes, in locations lacking the optical apparatus to make their light response sensitive to patterns within the direction of view [38,39]. By contrast, the ipRGCs that photo-entrain the mammalian circadian clock are located in the retina and thus, by nature, each samples light from a distinct portion of the visual scene. This arrangement has significance for the light-evoked activity in the SCN, where many neurons respond differently according to the location of a visual stimulus [4]. The obvious way in which spatial averaging can occur then is through temporal integration. Over time, head and eye movements will ensure that each ipRGC will experience large regions of the visual field. The poor temporal resolution of melanopsin phototransduction [40,41,42] could ‘blur’ responses to higher spatial frequency patterns such that maintained firing rate of individual retinal ganglion cells reflects light over a relatively large portion of the scene. However, it seems likely that processes within the SCN network also contribute to the necessary spatiotemporal integration [43]. Natural variations in change of view would probably be insufficient to allow an individual ipRGC to provide a faithful representation of the whole visual scene e.g., ipRGCs in dorsal retina would always be biased towards encoding light from the lower visual field. Moreover, time averaging within the circadian resetting mechanism would not be sufficient to overcome any influence of visual contrast on the maintained activity of ipRGCs [16]. Some feature of SCN physiology/anatomy may therefore be invoked to explain effective spatial integration for coarse patterns such as the zenith and horizontal spots applied here.

Our data support the continued use of irradiance as a simple and effective light metric for circadian biology. Thus, none of our patterned stimuli refute the parsimonious hypothesis that phase shift amplitude is well predicted by scene irradiance. This is an important conclusion as the irradiance metric provides a simple way to relate data from experimental conditions (in which spatial patterns are often explicitly minimised) to natural conditions in which patterns are unavoidable, and between scenes with very different light distribution. In principle, other metrics may provide a more reliable fit and our data are available to test alternative proposals. Here we test only one alternative hypothesis viz. that circadian responses scale according to the region of highest radiance. We find that this does not predict phase shift amplitude adequately.

A caveat to the conclusion that spatial patterns do not per se modulate circadian light responses is that they of course do make measures of irradiance dependent on the direction in which the detector is pointed. For this reason, it remains important to fully describe the spatial distribution of light in experimental and interventional studies, and to consider the subject’s predominant direction of view when making measurements [44].

## 4. Materials and Methods

### 4.1. Animals and Housing Conditions

All experiments were conducted in accordance with the UK Animals (Scientific Procedures) Act 1986 using adult male mice on a C57BL/6J background (Envigo, Belton, Loughborough, UK). Mice aged 10–12 weeks were singly housed in cages (300 × 130 × 115 mm) equipped with custom-made running wheels. Each wheel was fitted with an upright disposable cardboard running surface measuring 82 mm in diameter and 57 mm in width. Cages were maintained at approximately 22 °C in light-tight cabinets. Food and water were available ad libitum, and animal health and welfare were assessed daily. Before each light-exposure session, mice were entrained to a 12:12 h light–dark (LD) cycle for at least two weeks. Light stimuli were applied in a bespoke apparatus to which mice were transferred in darkness four hours after activity onset (designated ZT16). Light exposure lasted 15 min, after which animals were returned to their home cages in continuous darkness for a further 10 days. Mice reused for a subsequent exposure were then returned to the LD cycle for approximately 24 days before retesting, resulting in an interval of approximately five weeks between the two exposures within each paired comparison. Home cage wheel-running activity was recorded using a magnetically actuated sensor, logged using KitCollect and KitMonitor, with subsequent circadian analyses performed using KitAnalyzer (The Chronobiology Kit; Stanford Software Systems, Santa Cruz, CA, USA; version 1.0.0.1 for each program).

### 4.2. Light Exposure Apparatus and Stimuli

The light exposure apparatus comprised a ventilated, clear Perspex container measuring 15 cm in diameter and 15 cm in height, into which the mouse was introduced. The container was placed inside a larger chamber (75 × 75 × 110 cm) constructed of diffusive opal Perspex. For uniform and checkerboard illumination, the inner container served to restrict the mouse to the centre of the larger chamber, thereby limiting variation in viewing distance to the chamber walls as the animal moved. The outer chamber was externally illuminated with a violet-and-white LED system comprising 12 violet LED strips (Epistar 5050 LEDs, Hsinchu, Taiwan; 60 LEDs m^−1^; peak wavelength, 400 nm) and 12 LIFX A19 LED bulbs (B22, model LHA19B22UC10; LIFX, LiFi Labs Inc., San Francisco, CA, USA) arranged in three layers around the chamber to generate near-uniform, diffuse illumination throughout the chamber interior. The relative outputs of the violet and white LEDs were adjusted to approximate the relative pattern of excitation across mouse photoreceptor classes predicted for a clear-sky daylight spectrum recorded in Manchester, UK, in the late afternoon on 5 July 2013, at a solar elevation of approximately +30° [45]. Spectral irradiance measurements from 350 to 780 nm at 1-nm intervals (Figure 3) were analysed using the Animal Light Toolbox, based on the alphaopics package (v1.0.0; Git commit d47dad3). Mouse α-opic EDI values were calculated for M-cones, S-cones, rods, and melanopsin (Table 2). Melanopic EDI represents the illuminance of CIE standard illuminant D65 producing an equivalent melanopsin-weighted response to the measured spectrum, allowing standardized comparisons among lighting conditions [46,47].

An irradiance response curve for diffuse illumination was constructed initially as a dose finding exercise, by exposing each of 13 mice to a single irradiance across the range of 0.025 to 397.65 lx melanopic EDI. Irradiance levels producing sub-saturating phase shifts were then selected for comparisons between checkerboard stimuli and irradiance-matched uniform illumination. Checkers were produced by placing 4 layers of 0.9 neutral density gel (LEE Filters, Andover, UK; product no. 211) in a checkerboard pattern on the outside surface of the walls and ceiling of the large chamber. Basic trigonometric scaling was used to adjust mask dimensions in vertical and horizontal axes to correct for viewing angle relative to the centre of the arena and thus maintain a near common visual experience for the mouse across the whole above the horizon. We applied two checkers, both having a fundamental spatial frequency of 0.044 cpd when viewed from the centre of the arena, but differing in the fraction of the scene covered by the neutral density mask. In Checker 1, dark checks were three times wider than the bright checks yielding a 25% bright duty cycle and maximal radiance 12.32 log_10_(melanopsin-effective photons cm^−2^ s^−1^ sr^−1^). In checker 2, dark checks were seven times wider than the bright checks, yielding a 12.5% bright duty cycle while maintaining the same maximal radiance. Each was paired with diffuse illumination (no checkerboard mask) at the same measured irradiance (4.16 lx melanopic EDI and 2.10 lx melanopic EDI for checkers 1 and 2 respectively). Diffuse spot stimuli (approximately 16 cm in diameter) were produced using a single white LED bulb surrounded by violet LED strips placed outside the large chamber at a viewing distance of 75 cm. For these spot stimuli, the mouse container was repositioned away from the chamber centre as shown in Figure 2C to achieve the 75 cm viewing distance. The stimuli were presented either immediately above the horizon, with the lower edge of the patch aligned with the horizon such that the entire patch remained visible, or at the zenith (centre elevation = 90°).

### 4.3. Data Analysis

To allow for a within subjects experimental design, individual mice were exposed to either checker and its irradiance matched control or the diffuse spot at horizon or zenith in pseudo random order, with stimulus order balanced across animals to minimize order effects.

Phase shifts were determined by manually drawing lines of best fit through activity onsets during LD entrainment before stimulation and during constant darkness after stimulation. Each phase shift was measured as the displacement between the extrapolated lines at the time of stimulus presentation. Phase shifts were scored, from percentile-distribution actograms plotted on a 24-h time axis. For the irradiance response experiment, three blinded raters scored each actogram; the mean scores of the same two closely agreeing raters were used throughout. For the checkerboard and spot experiments, two blinded scores were averaged when they differed by <2 mm (approximately 35 min). For differences ≥ 2 mm, a third score was obtained and the two closest scores were averaged, or all three if the third was equidistant from the first two. One ambiguous actogram was independently reviewed by two additional scorers, and the two endorsed placements were averaged.

Phase shifts induced by checkerboard stimuli were compared with those induced by their irradiance-matched spatially uniform stimuli using two-tailed paired *t*-tests. Phase shifts induced by horizon and zenith spot stimuli were also compared using a two-tailed paired *t*-test.

For each comparison, we estimated the mean within-mouse difference in phase delay and its two-sided 95% confidence interval. Differences were calculated as checker minus the irradiance-matched uniform condition and as zenith minus horizon. Mean differences and confidence intervals were calculated using complete pairs only; all available observations, including unmatched observations, were retained for descriptive plotting and calculation of the observed condition means in Table 1. Confidence intervals were reported separately for each comparison without adjustment for multiplicity.

Statistical analyses and curve fitting were performed using custom code in MATLAB R2022a (The MathWorks, Inc., Natick, MA, USA) and GraphPad Prism 10 (GraphPad Software, Boston, MA, USA).

## Figures and Tables

**Figure 1:**
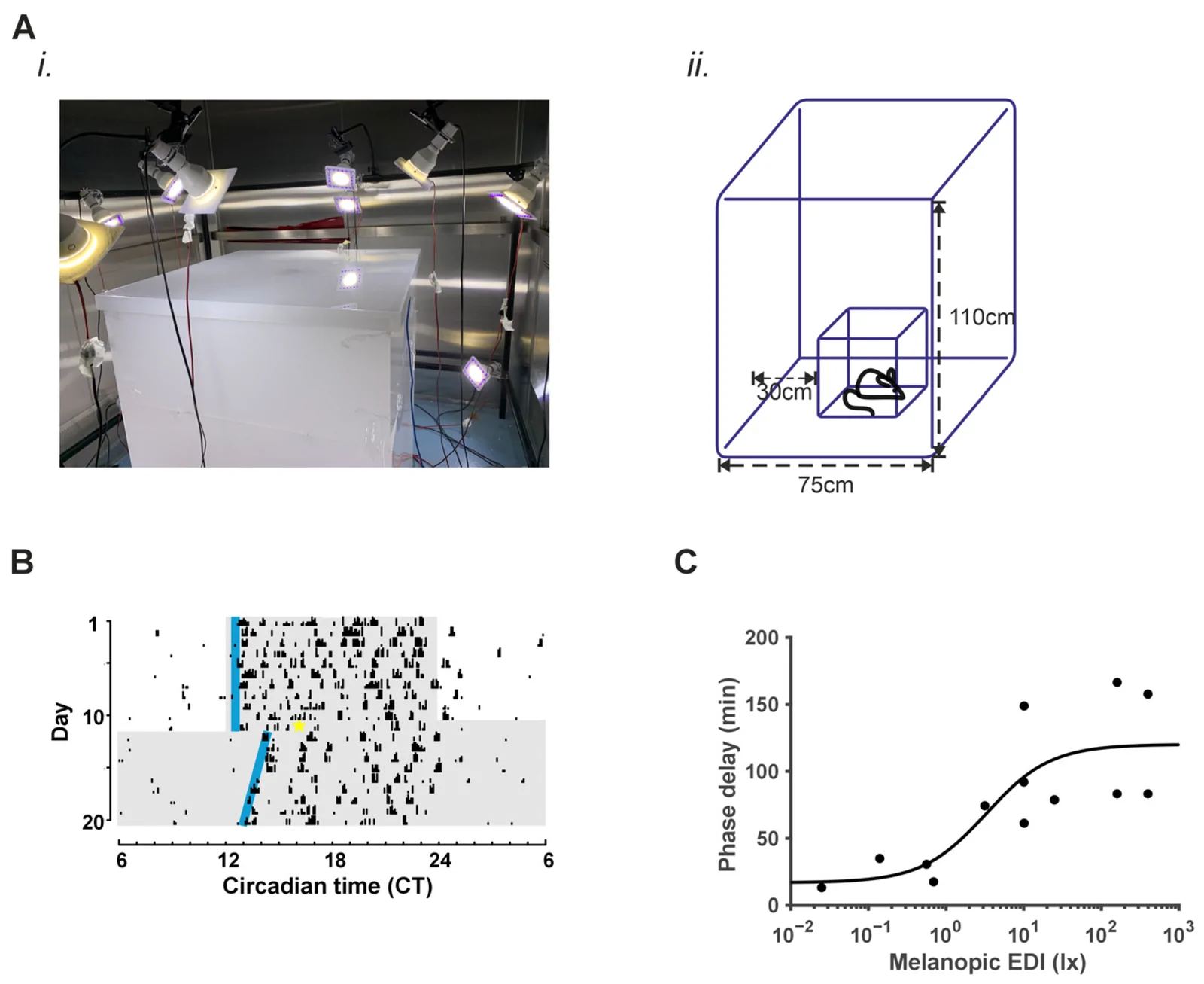
Light chamber and irradiance-dependent circadian phase resetting. (**A**) Photograph of exterior (**i**) of the light exposure apparatus constructed from diffusive opal Perspex and illuminated by violet and white LEDs arranged in three layers; and schematic (**ii**) showing dimensions and location of interior chamber for mouse. Purple outlines indicate the enclosures, and arrows indicate dimensions. (**B**) Representative cropped single-plotted actogram showing locomotor activity during entrainment to a 12:12 h light–dark cycle and subsequent constant darkness. Grey shading represents periods of darkness, the yellow star marks a 15 min light pulse and the blue lines indicate estimated times of activity onset before and after the light pulse, which were used to calculate phase shift amplitude. (**C**) Phase-delay response to uniform illumination as a function of scene irradiance fitted with a four-parameter logistic function (*n* = 13 mice; residual df = 9; R^2^ = 0.63; a half-maximal response at 3.29 lx melanopic EDI).

**Figure 2:**
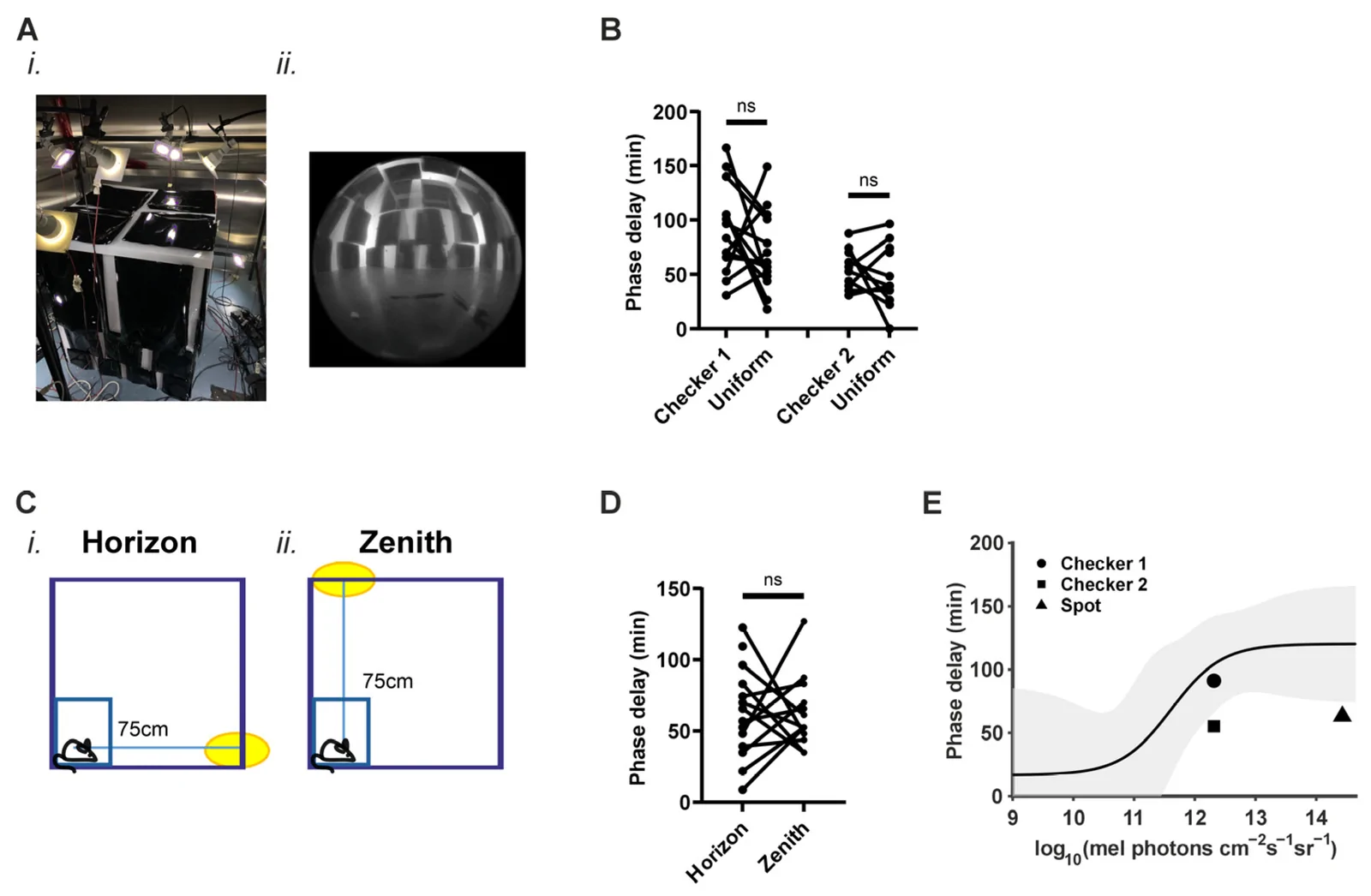
Spatial redistribution of light does not alter circadian phase resetting. (**A**) Photographs showing external (**i**) and internal (fish-eye) (**ii**) views of the light exposure apparatus set up for checkerboard by applying layers of neutral density gel. (**B**) Phase delays following exposure to checker 1 or 2 stimuli vs. irradiance matched uniform illumination. For Checker 1, 14 observations were plotted in each condition, and all 14 complete pairs were analysed. The mean paired difference (checker minus uniform) was 21.6 min (95% CI, −11.1 to 54.2 min; paired *t*-test, t(13) = 1.43, *p* = 0.18). For Checker 2, 10 checkerboard and 12 uniform observations were plotted, with 10 complete pairs analysed. The mean paired difference was 8.8 min (95% CI, −14.6 to 32.1 min; paired *t*-test, t(9) = 0.85, *p* = 0.42). (**C**) Schematics of a 16-cm-diameter light patch (yellow) presented either immediately above the horizon, with its lower edge aligned with the horizon so that the entire patch remained visible (**i**), or at the zenith ((**ii**); centre elevation = 90°). Purple outlines show the exposure apparatus, dark blue outlines show the interior chamber for mouse, and pale blue lines indicate viewing distances. (**D**) Phase delays induced by light at the two locations. All available observations are plotted (horizon, *n* = 14; zenith, *n* = 13); lines connect the 13 complete pairs used in the paired *t*-test. The mean paired difference (zenith minus horizon) was 3.0 min (95% CI, −22.8 to 28.9 min; paired *t*-test, t(12) = 0.26, *p* = 0.80). (**E**) Phase delay as a function of peak scene radiance for checker 1 (filled circle) and 2 (filled square) and spots at horizon or zenith (filled triangle) plotted against the curve fit ± 95% confidence interval (shading) for responses to uniform illumination (data replotted from Figure 1C). In panels (**B**,**D**), lines connect paired observations; ns, not significant (*p* > 0.05).

**Figure 3:**
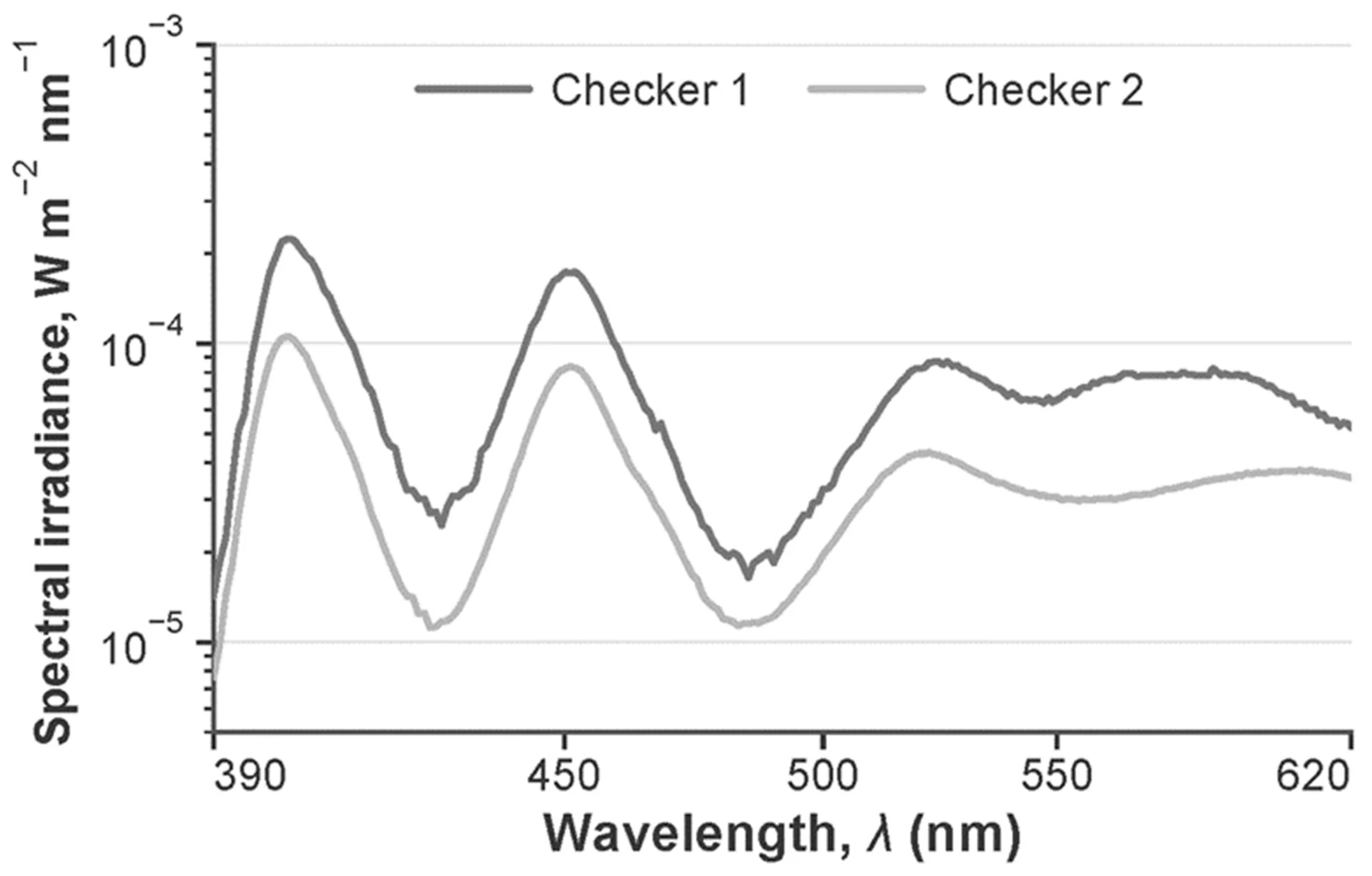
Spectral irradiance distributions of Checker 1 (grey) and Checker 2 (light grey) from 390 to 620 nm. Spectral irradiance is expressed in a logarithmic scale.

**Table 1:** Phase delays predicted from the peak-radiance response curve compared with observed phase delays.

	Peak Radiance, log_10_(Melanopsin-Effective Photons cm^−2^ s^−1^ sr^−1^)	Predicted Phase Delay, Min, Mean (95% CI)	Observed Phase Delay (Min)
Checker 1	12.32	104.2 (66.1–142.2)	91.3
Checker 2	12.32	104.2 (66.1–142.2)	55.2
Horizon	14.35	120.1 (74.7–165.5)	63.2
Zenith	14.43	120.1 (74.5–165.7)	62.7

Observed means were calculated using all available observations (Checker 1, *n* = 14; Checker 2, *n* = 10; horizon, *n* = 14; zenith, *n* = 13).

**Table 2:** Mouse α-opic equivalent daylight illuminance under different lighting conditions.

Lighting Condition	α-Opic Equivalent Daylight Illuminance (EDI; lx)			
	M-Cone-Opic	S-Cone-Opic	Rhodopic	Melanopic
Daylight	11,409.06	7075.76	11,319.28	11,131.75
Checker 1	4.24	1.98	4.16	4.16
Checker 2	2.11	0.82	2.08	2.10

## Data Availability

The data underlying the figures and reported statistical comparisons are openly available in the Open Science Framework (OSF) at https://doi.org/10.17605/OSF.IO/M2QZ3.

## Abbreviations

The following abbreviations are used in this manuscript:

SCN	Suprachiasmatic nuclei
ipRGCs	Intrinsically photosensitive retinal ganglion cells
LD	Light–dark
ZT	Zeitgeber time
EDI	Equivalent daylight illuminance
cpd	Cycles per degree
CI	Confidence interval
CIE	International Commission on Illumination
